# A glimpse into the CyberKnife robotic radiosurgery system: technical specifications and developments during thirty years

**DOI:** 10.3389/fonc.2026.1871489

**Published:** 2026-06-29

**Authors:** Payam Samadi Miandoab, Yansong Wu, Hui jun Xu, Jun Yang, Gang Ren, Bo Liu, Chao Wang, Gaolong Zhang, Shouping Xu

**Affiliations:** 1 National Cancer Center/National Clinical Research Center for Cancer/Cancer Hospital, Chinese Academy of Medical Sciences and Peking Union Medical College, Beijing, China; 2 School of Physics, Beihang University, Beijing, China; 3 Department of Engineering Physics, Tsinghua University, Beijing, China; 4 Department of Radiation Oncology, Foshan Fuxing Chancheng Hospital, Foshan, China; 5 Department of Radiation Physics Solution, Drexel University, Phiadelphia, PA, United States; 6 Department of Radiotherapy, Peking University Shougang Hospital, Beijing, China; 7 Image Processing Center, Beihang University, Beijing, China

**Keywords:** CyberKnife system, image-guided therapy, precision assessments, robotic radiosurgery system, stereotactic body radiation therapy

## Abstract

The CyberKnife system is a state-of-the-art technology widely used in the treatment of cancer. It incorporates a 6 MV X-band linear accelerator mounted on a six-degree-of-freedom (6DOF) robotic arm, combined with an image-guidance platform, to deliver high-dose stereotactic body radiation from a variety of unique angles with sub-millimeter accuracy to the targets in the body. As a frameless and semi-automated treatment, CyberKnife also enables patients to breathe normally during treatment while the robotic arm dynamically tracks and corrects their motion. This review aims to provide an overview of the evolution of the CyberKnife system from its introduction in 1994 through 2026, summarize target-tracking strategies, assess accuracy in both static and moving targets, and highlight its clinical applications, including re-irradiation settings, dose prescriptions and survival outcomes. Additionally, the advantages, challenges, and recent advances of the CyberKnife system are discussed to provide a comprehensive perspective on its role in modern radiotherapy.

## 1 Introduction

Radiotherapy uses high-energy rays to destroy cancer cells (1). Brachytherapy places the radiation source inside or near the tumor, while external beam radiation therapy (EBRT) delivers radiation from outside the body using a linear accelerator. The main goal of EBRT is to accurately conform the radiation dose to the tumor volume while minimizing exposure of surrounding healthy tissues (2, 3). In this regard, Swedish neurosurgeon Dr. Lars Leksell introduced the stereotactic head frame in 1951 to immobilize patients during intracranial treatments, thereby ensuring highly accurate dose delivery (4). This innovation laid the foundation for stereotactic radiosurgery (SRS) and stereotactic body radiotherapy (SBRT), which have since become widely adopted techniques in modern radiation oncology (1, 2). Today, advanced EBRT often incorporates image-guided radiation therapy (IGRT), which uses advanced imaging throughout treatment to improve targeting accuracy. These technologies enable the delivery of high radiation doses to tumors in either a single session (SRS) or a limited number of fractions referred to as stereotactic radiation therapy (SRT) (1, 4). While these techniques can be performed on widely-available C−arm linear accelerators (Linacs) using systems such as surface guidance or ExacTrac, several specialized systems—such as TomoTherapy, CyberKnife^®^, and Gamma Knife—offer specific advantages for certain indications (5). Among these, the CyberKnife system is particularly notable for its ability to perform highly accurate real-time motion tracking, enabling sub-millimeter targeting accuracy for moving targets. While previous reviews have provided comprehensive technical overviews of the CyberKnife system (6, 7), the present work aims to synthesize technical evolution with clinical evidence across three decades, with particular emphasis on comparative motion management strategies and recent technological advances that distinguish current-generation systems from their predecessors, as well as emerging applications such as re-irradiation. Accordingly, this study is organized around four primary objectives: 1) to review the evolution and component advancements of the CyberKnife system between 1987 and 2026; 2) to describe target-tracking techniques and assess their accuracy for both stationary (rigid) and moving targets; 3) to highlight clinical applications alongside the benefits and limitations; and 4) to present the most recent advances in the system.

## 2 History of the CyberKnife system

**Figure 1:**
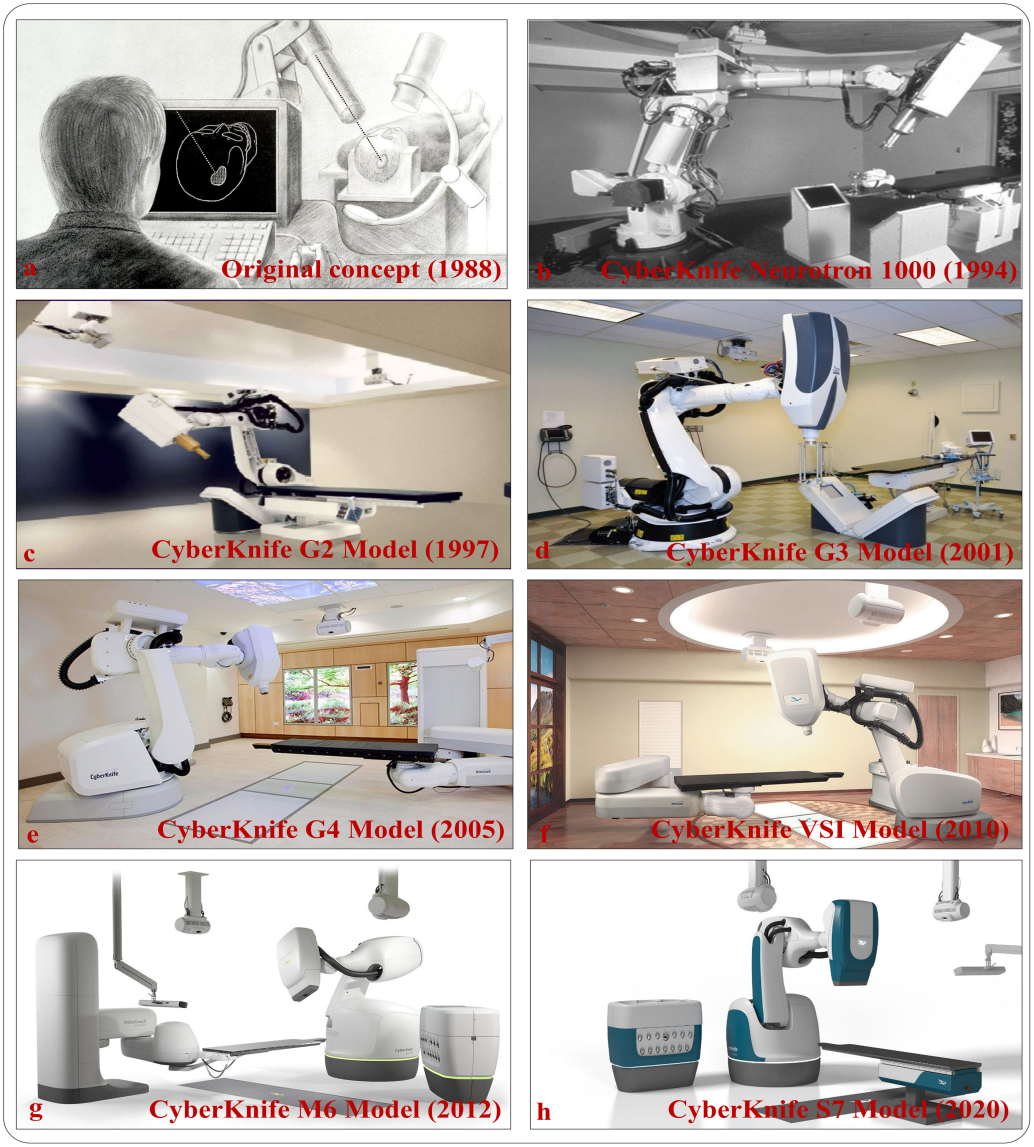
The CyberKnife accuray product generation from 1987 to 2020. **(a)** Original Concept (1988), **(b)** CyberKnife Neurotron 1000 (1994), **(c)** CyberKnife G2 system (1997), **(d)** CyberKnife G3 system (2001), **(e)** CyberKnife G4 system (2005), **(f)** CyberKnife VSI system (2010), **(g)** CyberKnife M6 system (2012), and h) CyberKnife S7 system (2020).

As Scott Belsky once noted, *“It is not about ideas, it is about making ideas happen.”* This sentiment reflects the origins of the CyberKnife system. The concept was first introduced by Professor John R. Adler, a neurosurgeon at Stanford University, who envisioned a frameless radiosurgical device (**Figure 1a**) after being trained on the framed SRS technology by Dr. Leksell. Adler, along with Peter and Russell Schonberg from the Schonberg Research Corporation, developed the first CyberKnife^®^ robotic system in 1988 (8). In 1994, a collaboration between Stanford University and Accuray Incorporated (Sunnyvale, CA, USA) led to the creation of the CyberKnife prototype, known as the Neurotron 1000. That same year, on June 8, 1994, the first patient—a case of brain metastases—was successfully treated using this system. Between 1999 and 2001, the U.S. Food and Drug Administration (FDA) granted approval for the CyberKnife to treat both intracranial (in the head and upper spine) and extracranial tumors. Since then, Accuray has continued to refine the technology, releasing six successive generations of the CyberKnife system by 2020 (9). The original prototype, the Neurotron 1000, was installed at Stanford University Medical Center between 1994 and 2000 (**Figure 1b**) (9, 10). The second generation of the CyberKnife system (**Figure 1c**) was launched in 1997, featuring key upgrades to both the robotic arm and imaging system. Specifically, the GMFanuc robotic arm was replaced with a KUKA robot arm (KUKA Systems GmbH, Augsburg, Germany), and the fluoroscopic screen camera was substituted with flat-panel amorphous silicon detectors, improving imaging quality and reliability (9). The third-generation CyberKnife (G3, **Figure 1d**), released in 2001, integrated enhanced image-guided tracking algorithms, enabling more precise high-dose radiation delivery for both static and moving targets. This generation features innovative screw-free tracking capabilities, including six-dimensional (6D) skull tracking, XSight^®^ spine tracking, and the Synchrony^®^ respiratory tracking system (9). By 2005, the fourth-generation CyberKnife (G4, **Figure 1e**) became available, equipped with an automated collimator exchange table. This improvement streamlined treatment delivery by enabling efficient switching between collimators during therapy, thereby reducing overall treatment time (9). The CyberKnife VSI system, introduced in 2010 (**Figure 1f**), incorporated several significant upgrades. These included a 6D robotic treatment couch for precise patient positioning, a high-resolution amorphous silicon flat-panel detector (1024 × 1024 pixels), an increased dose rate of 800 monitor units (MUs) per minute, and an additional shielding ring to minimize radiation leakage. It also featured the Iris^™^ variable aperture collimator system, the Xchange^™^ collimator table, and a fiducial-less lung tracking capability (6, 9). In subsequent years, the CyberKnife M6 system (**Figure 1g**) was released, introducing significant hardware advancements (i) the digital signal platform, (ii) improved treatment efficiency and reduced delivery time, (iii) integration of a modern Kuka robotic arm (KR300 R2500 Ultra), and (iv) the capability to employ a multi-leaf collimator (MLC) for enhanced beam shaping (7, 9). The latest generation, the CyberKnife^®^ S7^™^ System, was launched on June 17, 2020 (**Figure 1h**). This platform introduced several innovations designed to streamline the installation, planning, and delivery of radiation beams to target volumes. The system integrates the Synchrony^®^ module with the VOLO^™^ optimizer, which enhances reverse planning by leveraging the planning capabilities of the previous optimizer. Additionally, a real-time artificial intelligence (AI)-based predictive model was incorporated, allowing external beam delivery for SRS and SBRT treatments in as little as 15 minutes (11). Notably, the first patient treated with the S7 system was at Geisinger Wyoming Valley Medical Center. Since its introduction, the CyberKnife platform has been used to treat an estimated 400, 000 patients with tumors in the brain, spine, lung, liver, prostate, and other extracranial sites (7, 11).

## 3 CyberKnife system components and developments

The CyberKnife system comprises nine subsystems—robotic manipulation (including the treatment manipulator, coordinate systems and workspace calibration, treatment paths and node properties, proximity detection and collision avoidance, the Xchange table and tool mounting calibration, and the RoboCouch), the treatment head, secondary collimation devices (including 12 fixed cone collimators, the Iris collimator, and the InCise MLC), X-ray and optical imaging systems, target tracking and localization techniques for treatment delivery (such as registering live X-ray images with digitally reconstructed radiographs (DRRs) and real-time respiratory motion tracking), along with image registration and segmentation tools for treatment planning (including multimodality image import and registration, automated segmentation, and re-irradiation support). Additionally, treatment planning (radiation dose calculation and optimization algorithms), data management, and connectivity systems are incorporated, with detailed descriptions available in the literature (6, 7), While the Supplementary Material highlights the significant technological advances achieved over the past thirty years.

## 4 Target tracking methods

**Figure 2:**
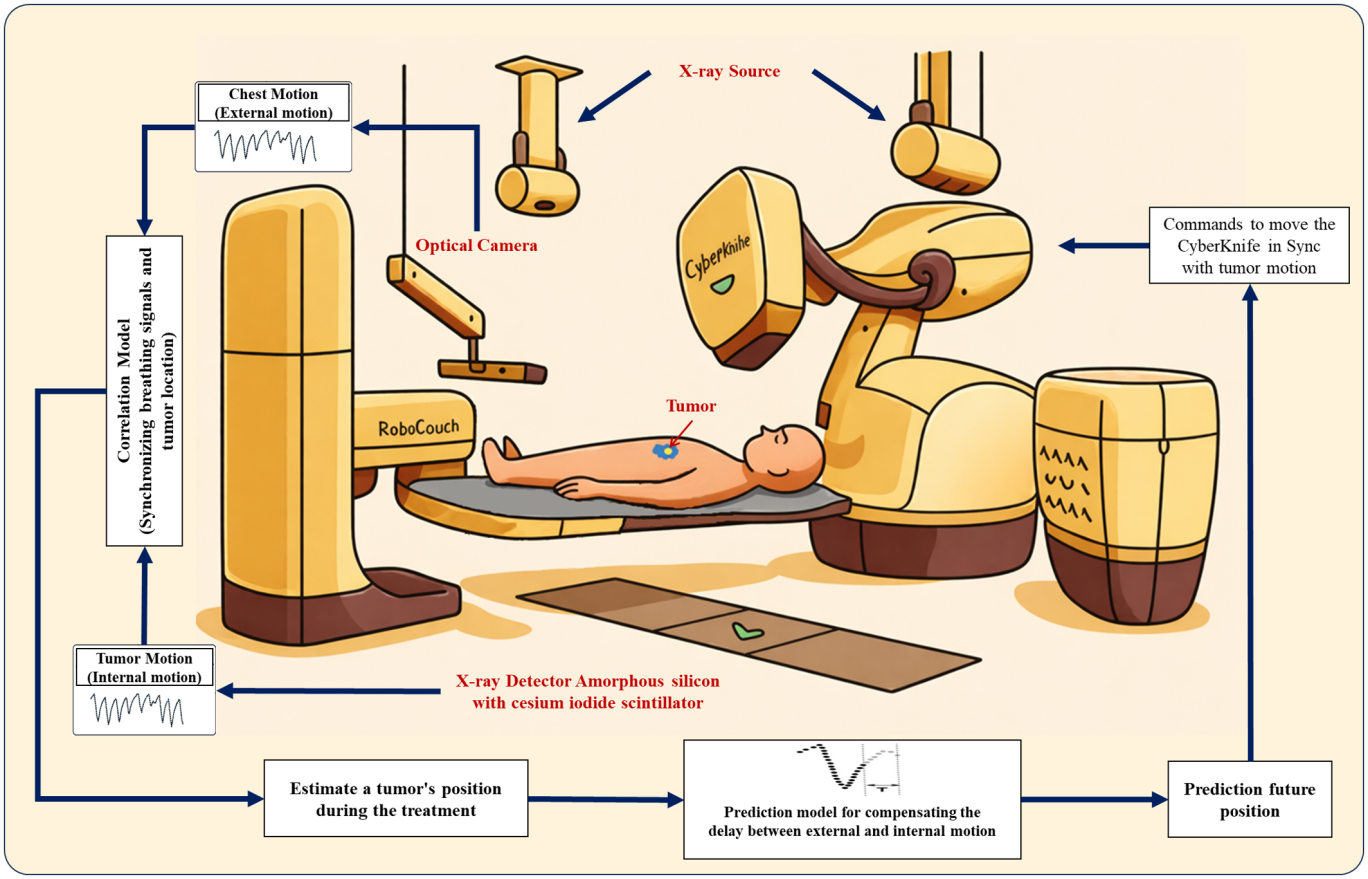
A schematic block diagram of the SRTS in the CyberKnife system.

To achieve accurate target localization and tracking, the CyberKnife system utilizes advanced image-guided algorithms that fall into two primary categories. The first group involves live X-ray registration with DRRs for target localization, including 6D skull tracking for intracranial and upper cervical spine or head and neck targets using skeletal features; fiducial marker tracking for soft-tissue targets such as the prostate, liver, pancreas, and breast; Xsight^®^ spine tracking for targets within or fixed relative to the spine; Xsight^®^ spine prone tracking for fiducial-free spinal radiosurgery in face-down patients; Xsight^®^ lung tracking for soft-tissue targets without fiducial markers; and the lung-optimized treatment (LOT) method, an advanced non-invasive robotic technique that uses real-time tumor tracking via 0-view, 1-view, or 2-view imaging to eliminate implanted fiducial markers and reduce patient risk (6, 7). The second group processes real-time respiratory motion tracking using the Synchrony Respiratory Tracking System (SRTS) and the InTempo method (6, 7). Notably, real-time respiratory tracking enables continuous adjustment of external beams to follow tumor respiratory motion. The core principle of the SRTS correlation model is the synchronization of respiratory patterns with tumor position (6, 7). The CyberKnife system incorporates a stereo camera that tracks three red LED markers on the patient’s body at 26 Hz. Two orthogonal diagnostic X-ray source/detector pairs are employed during pre-treatment to determine the tumor location, typically identified by fiducial seeds visible on radiographic images (**Figure 2**). Multiple images are acquired to determine the tumor’s position and correlate it with the motion of the external LED markers. The respiratory model is built during the treatment setup. After setup, the CyberKnife respiratory model estimates internal tumor motion from the LED markers’ external motion. To maintain sub-millimeter accuracy, this correlation model is periodically updated with fresh X-ray images every 30–60 seconds, using the most recent 2 minutes of data via a first-in, first-out (FIFO) method (6, 7). However, system latency—arising from data acquisition and robotic response—can influence the precision of radiation delivery. To address this, a prediction model is applied to compensate for latency by estimating the discrepancy between predicted motion and actual tumor position. Depending on the CyberKnife system version, this approach is used to correct for latencies of approximately 115–200 ms during radiotherapy (6, 7).

## 5 Precision assessments

**Figure 3:**
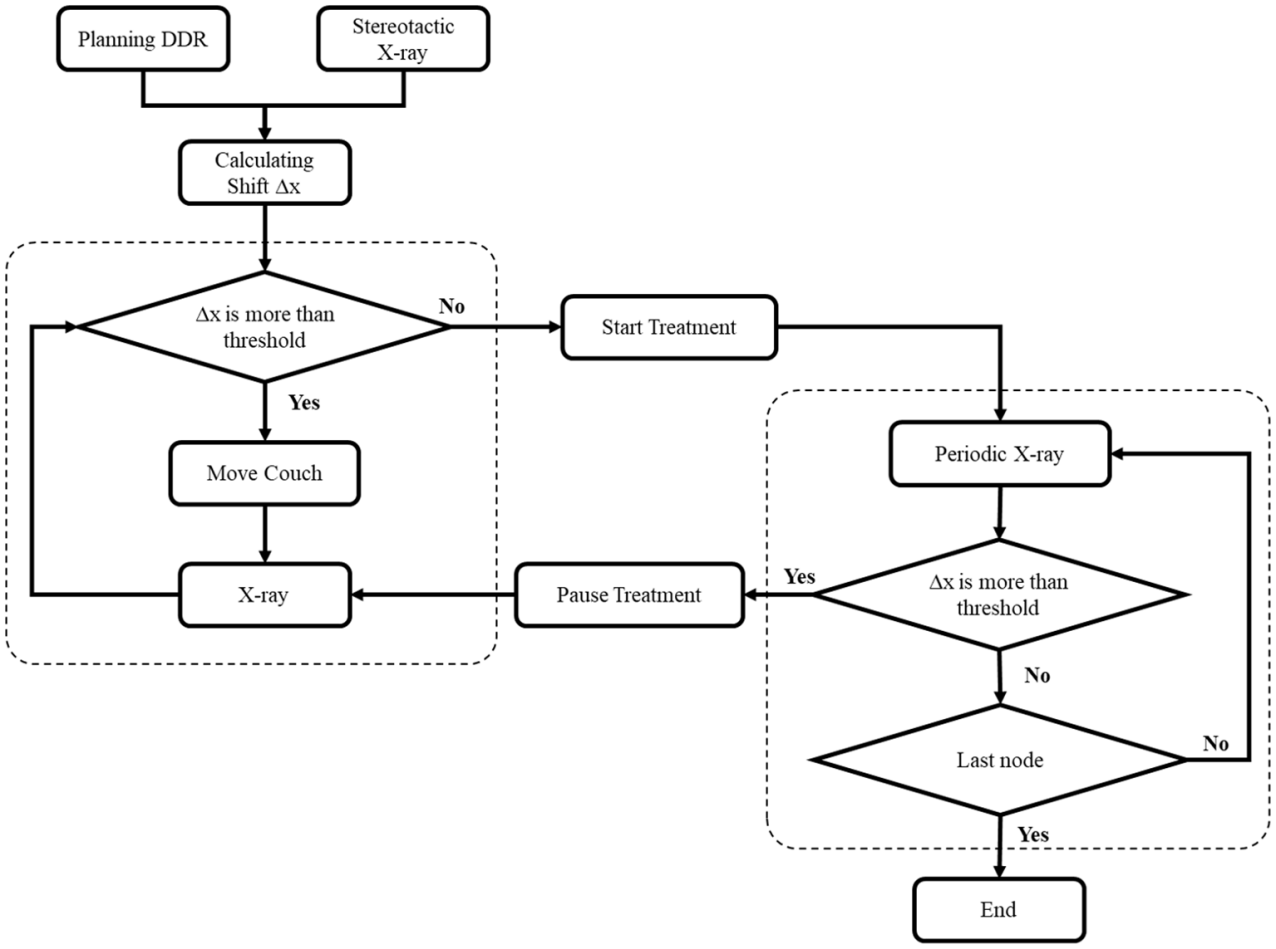
Workflow of the patient setup and verification in the CyberKnife system.

In the CyberKnife system, patient setup and verification are performed using an image-guidance system that registers live X-ray images with DRRs from the treatment plan to detect and correct positional shifts (6, 7). If the detected shift exceeds a threshold or erratic motion is observed, treatment is paused, and the couch is automatically adjusted. The process involves two steps: an initial manual alignment and a precise automatic alignment to ensure accurate dose delivery (**Figure 3**). Overall, this dual-step image-guided process has established CyberKnife as one of the most accurate robotic platforms for patient alignment, consistently achieving sub-millimeter precision in clinical practice. However, reliance on bony landmarks and fiducials can still introduce residual uncertainties, particularly in deformable organs. In the authors’ view, there remains a need for a more universal image-guidance method that is not limited to specific anatomical regions (such as the head and spine) but can also be reliably applied to pelvic and other deformable sites.

Dose calculation in the CyberKnife system is performed using either the Type A algorithm (Ray Tracing (RT), finite-size pencil beam (FSPB), and FSPB with lateral scaling option (FSPB_LS)) or the Type B algorithm (Monte Carlo (MC)) (7, 11). Technically, both algorithms can achieve comparable accuracy under certain conditions; however, the Type B algorithm (MC) provides more reliable dose calculation for lung nodules or near tissue–air interfaces, albeit at the expense of increased computation time (12–15). Numerous studies have collectively evaluated the performance and accuracy of various CyberKnife dose calculation algorithms, including RT, FSPB, FSPB_LS, and MC, across different anatomical sites and clinical conditions (12–15). Overall, MC is the most accurate dose calculation algorithm for CyberKnife, particularly in complex or heterogeneous tissues such as the lung, thoracic spine, and multi-target brain treatments. Other algorithms, such as FSPB and RT, are faster but less accurate in challenging conditions. FSPB_LS improves upon FSPB, yet MC remains the most reliable for final verification and planning in heterogeneous regions. Collectively, the accumulated evidence clearly supports MC as the preferred dose calculation algorithm for high-precision CyberKnife treatments in heterogeneous tissues. However, the substantial increase in computational time remains a notable limitation in high-volume centers, illustrating an ongoing trade-off between dosimetric accuracy and clinical workflow efficiency.

A large advantage of CyberKnife is its proven ability to manage respiratory motion through its Synchrony^®^ system, which provides continuous, real-time robotic tracking during free breathing (6, 7). While this remains a benchmark, particularly for hypofractionated treatments, several state-of-the-art Linac-based technologies have emerged, each offering a distinct approach to motion management (16–28). The VERO system pioneered Linac-based tracking using a gimbaled Linac head, achieving geometric and dosimetric accuracy comparable to that of the CyberKnife system. However, as a specialized non-robotic platform, its clinical availability remains limited today (16–18). In contrast, more widely available solutions, such as Varian’s triggered imaging and respiratory gating (e.g., RPM system), use external surrogates to gate the beam. While non-invasive and accessible, this method reduces the duty cycle—thereby prolonging treatment time and increasing the risk of interplay effects. In addition, its accuracy may be limited by hysteresis between external and internal motion as well as baseline drift during treatment when correlation model updates are not performed (20, 21). Technologies like Kilovoltage Intrafraction Monitoring (KIM) offer a hardware-efficient alternative by using kV imaging on conventional Linacs to estimate 3D target position for adaptive radiotherapy. However, this typically involves a higher imaging dose and often relies on gating or intermittent corrections rather than the continuous beam following of Synchrony (22–26). The most advanced imaging-based approach is the MR-Linac, which provides superior soft-tissue visualization without fiducials for real-time gating and adaptation. In principle, real-time motion monitoring could be further enhanced by synchronizing high–temporal resolution 3D cine MRI for internal 6D motion assessment—potentially including deformation—with surface-guided radiotherapy for external 6D motion monitoring. Despite its advantages, the MR-Linac faces higher system latency (~300–350 ms vs. CyberKnife’s ~115 ms), high costs, and reliance on gating rather than full dynamic tracking. Additionally, some studies have reported that this higher system latency can lead to greater prediction errors in target motion management (29–31).

Appropriate precision evaluation is essential in the CyberKnife system to quantify uncertainties, assess their impact on dose distribution, and determine suitable planning target volume (PTV) margins. Multiple sources of uncertainty must be considered, including uncorrected target deformation, systematic rotational errors, and motion-modeling inaccuracies. These errors are strongly influenced by tumor shape, location, respiratory motion amplitude, and the placement of external LED markers (32–34). Routine quality assurance is critical for maintaining system reliability. End-to-end (E2E) testing evaluates the entire treatment chain using anthropomorphic phantoms and typically requires overall geometric accuracy of less than 0.95 mm (43–45). Delivery quality assurance (DQA) verifies plan deliverability through point-dose measurements and 2D dose analysis, commonly aiming for <3% dose difference and >90% gamma passing rate in high-precision SRS/SBRT (35). System latency, which arises from image acquisition, prediction modeling, and robotic response, is another key factor affecting tracking accuracy (32–34).

Tracking performance varies depending on the CyberKnife generation, correlation and prediction models, tumor site, and patient-specific characteristics. Therefore, CyberKnife tracking accuracy can be broadly categorized into two groups: stationary or semi-stationary targets (e.g., brain and spine), and dynamic motion tracking for moving targets (e.g., lung and liver tumors). The prostate, which exhibits well-documented intrafraction drift and rotation, is more appropriately considered a semi-stationary target. **Table 1** summarizes the reported tracking accuracy for both categories, with 3D radial error calculated as the root-sum-square of the deviations in each direction.

**Table 1:** The target localization and tracking accuracy in different CyberKnife systems.

	Study by	CyberKnife version	Tracking method †	Clinical accuracy mean ± SD (mm)			
				SI direction	AP direction	LR direction	3D radial
Stationary Lesions	Yu et al. in 2004 (79)	G3 CyberKnife System	Fiducial Tracking Method	-0.22 ± 0.44	-0.28 ± 0.33	-0.26 ± 0.25	0.68 ± 0.29
	Antypas and Pantelis in 2008 (80)	G4 CyberKnife System	6D Skull Tracking Method	-0.18 ± 0.11	0.24 ± 0.03	-0.04 ± 0.35	0.44 ± 0.12
	Antypas and Pantelis in 2008 (80)	G4 CyberKnife System	Fiducial Tracking Method	-0.16 ± 0.17	0.08 ± 0.17	-0.07 ± 0.12	0.29 ± 0.10
	Antypas and Pantelis in 2008 (80)	G4 CyberKnife System	Xsight^®^ spine Tracking Method	-0.18 ± 0.25	-0.06 ± 0.21	-0.18 ± 0.43	0.53 ± 0.16
	Nano et al. in 2020 (81)	VSI CyberKnife System	6D Skull Tracking Method	-0.10 ± 0.06	0.15 ± 0.03	-0.05 ± 0.06	0.19 ± 0.09
	Nano et al. in 2020 (81)	VSI CyberKnife System	Fiducial Tracking Method	-0.11 ± 0.08	-0.06 ± 0.03	-0.09 ± 0.05	0.15 ± 0.10
Moving Lesions	Samadi et al. in 2022 (33)	G3 CyberKnife System	Fiducial Tracking Method	0.57 ± 1.42	0.56 ± 1.33	0.59 ± 1.44	–
	Jung et al. in 2015 (82)	VSI CyberKnife System	Fiducial Tracking Method	0.36 ± 0.39	0.15 ± 0.62	0.15 ± 0.64	–
	Jung et al. in 2015 (82)	VSI CyberKnife System	Xsight^®^ Lung Tracking Method	0.38 ± 0.54	0.14 ± 0.37	0.13 ± 0.18	–
	Winter et al. in 2015 (83) *	VSI CyberKnife System	Fiducial Tracking Method	1.24 ± 1.61	0.65 ± 0.85	0.75 ± 1.02	1.79 ± 1.16
	Nano et al. in 2020 (81)	VSI CyberKnife System	Xsight^®^ spine Tracking Method	0.09 ± 0.22	0.04 ± 0.09	-0.08 ± 0.05	0.12 ± 0.10
	Nano et al. in 2020 (81)	VSI CyberKnife System	Xsight^®^ Lung Tracking Method	-0.18 ± 0.05	0.13 ± 0.04	0.22 ± 0.11	0.31 ± 0.13
	Samadi et al. in 2023 (32)	VSI CyberKnife System	Xsight^®^ Lung Tracking Method	0.44 ± 1.16	0.22 ± 0.90	0.23 ± 0.90	–
Mechanical Accuracy	Yang et al. in 2012, the mean mechanical accuracy was 0.12 mm for G4 Model (84).						
	Okamoto et al. in 2016, the mean ± SD mechanical accuracy was 0.07 ± 0.35 mm, 0.01 ± 0.35 mm, and -0.09 ± 0.36 mm in the SI, LR, and AP directions for the VSI Model (85).						
	Kilby et al. in 2020, the mean mechanical accuracy 0.06 mm for M6 and S7 Models (7).						

† Since the release of CyberKnife System 11.2 and Precision 3.3, Accuray Inc. has updated the terminology used for tracking methods on both the system console and in official documentation.

The maximum targeting accuracy reported by the Accuray Company for the Xsight^®^ spine tracking method was 0.95 mm.

The maximum targeting accuracy reported by the Accuray Company for the Synchrony^®^ Respiratory Tracking System was 1.5 mm.

The end-to-end (E2E) test result was between 0.3 and 0.7mm.

* Absolute mean value was reported for clinical accuracy.

SI, Superior-Inferior, AP, Anterior-Posterior, LR, Left -Right.

Several studies have provided robust methodologies for error analysis and PTV margin determination (32, 34, 36, 37). For real-time tumor tracking with CyberKnife, recommended PTV margins are generally 3–5 mm (commonly 3–4 mm) for lung, 4 mm for liver, and 4–5 mm for pancreas, reflecting differences in deformation and motion irregularity. In contrast, C-arm linac-based SBRT, which typically relies on gating, breath-hold, or ITV-based approaches rather than continuous real-time tracking, often requires larger margins of 5–10 mm (or ITV plus 3–5 mm setup margin) for lung and liver targets (32, 34, 36–39). In summary, the accumulated evidence confirms that CyberKnife’s real-time tracking enables significantly smaller PTV margins compared to conventional linac-based gating or ITV approaches, particularly for moving targets. This margin reduction has been a key driver in the adoption of CyberKnife for hypofractionated treatments. Nevertheless, in the authors’ view, the need for invasive fiducials in some cases and the reliance on the quality of the correlation model continue to represent important limitations, particularly when benchmarked against emerging fiducial-less MR-guided platforms.

## 6 Clinical application overview

The CyberKnife system is a radiosurgery platform capable of treating tumors throughout the body (40–45). This overview, although not exhaustive, highlights key treatment considerations—including primary and secondary malignant lesions (metastases), benign lesions, and functional disorders—and presents the results in a concise and practical form, as a comprehensive review of all available studies is beyond the scope of this article. In clinical practice, tumors selected for CyberKnife treatment generally need to meet a few key criteria (1): they should not be too large for SBRT, (2) they should maintain a safe distance from, or allow for sufficient sparing of, adjacent organ-at-risk (OARs) such as the bowel, esophagus, and bladder, and (3) their boundaries must be clearly definable. Although a certain distance from critical OARs is preferred (the second criterion), tumors in proximity or even in direct contact with these structures are not excluded. In such cases, treatment remains feasible using a hypofractionated regimen with adjusted (lower) dose per fraction and strict adherence to OAR dose constraints. Pancreatic and prostate cancers are typical examples of this approach.

Although a comprehensive case-by-case dosimetric comparison is beyond the scope of this review, multiple studies provide important insights into the relative performance of CyberKnife and modern linac-based SBRT (46–51). The PACE-B prostate trial found that despite higher doses to urinary structures, CyberKnife patients experienced lower urinary toxicity and reduced rectal exposure. While this finding is intriguing, it should be interpreted with caution, as potential confounding factors—such as differences in patient risk profiles, center experience, and fractionation schedules—may contribute to the observed differences in toxicity. Therefore, further investigation is warranted before attributing the outcome directly to the delivery platform (47). For single brain metastases (n=10), RapidArc offered significantly shorter treatment time, lower integral brain doses, lower V_12_ (3.27 vs. 4.09 cc), and comparable conformity, while CyberKnife provided superior dose gradient (4.52 vs. 8.6) (48). In renal cell carcinoma (n=10), VMAT and proton therapy achieved equivalent or superior target coverage with better OAR sparing than CyberKnife (49). For early-stage non-small cell lung cancer (NSCLC) (n=106), no significant differences were found in local control, survival, or toxicity between platforms (50). For laryngeal SBRT (n=10), linac VMAT achieved similar quality with superior thyroid sparing and <1/3 MUs of CyberKnife (51).

The CyberKnife system delivers high-dose, exact radiation in a few fractions to a limited number of liver metastases (typically ≤5 lesions, ≤6 cm in size) in patients not suited for surgery, offering excellent local control rates of 70-100% at one year and 60-90% at two years with minimal severe toxicity (under 5%) (45). In a cohort of 106 medically inoperable, early-stage non-small cell lung cancer patients with predominantly stage IA/B disease, SBRT delivered via the CyberKnife system—utilizing real-time tumor tracking and risk-adapted fractionation schedules (3×17 Gy, 5×11 Gy, or 8×7.5 Gy)—achieved satisfactory outcomes with actuarial local control rates of 88% at 2 years and 77% at 3 years, a median disease-free survival of 27 months, and overall survival rates of 77% at 2 years and 56% at 3 years, all while maintaining low toxicity profiles characterized by mild acute side effects and only 4% late effects (44). **Table 2** provides additional examples of survival rates for various tumor types treated with the CyberKnife system. Overall, survival rates following CyberKnife treatment vary considerably, depending on multiple clinical and biological factors. These include tumor size and location, histology, disease stage, patient health status, prior treatments, prescribed dose, number of fractions, and tumor biology/genetics (52, 53).

**Table 2:** Summary of clinical outcomes and treatment parameters for CyberKnife SRS/SBRT across tumor sites.

Study by	Prescribed Dose (Gy)/Fraction	Summary of Clinical Outcomes
Ung-Kyu Chang et al. in 2009 (86)	16–39 Gy in 1–5 fractions	In a study of 129 patients with 167 spinal metastases treated with CyberKnife, 6-month follow-up showed a pain relief rate of 91% and radiological control in 90% of lesions (75/83), with zero treatment-related radiation injury.
JY Que et al. in 2014 (87)	26–40 Gy in 5 fractions	In 22 patients with unresectable huge HCC (≥10 cm) treated with SBRT, the objective response rate was 86.3%, 1-year local control was 55.6%, median overall survival was 11 months, SBRT dose was the most significant prognostic factor (p = 0.0072), and acute toxicities were mild and well tolerated.
Zhi-Yong Yuan et al. in 2014 (88)	39–54 Gy in 3–7 fractions	In 57 patients with 80 liver metastases (≤4 lesions, <6 cm) treated with CyberKnife SBRT, 1-year and 2-year local control rates were 94.4% and 89.7% respectively, median overall survival was 37.5 months (72.2% 2-year OS in favorable primary tumors vs. 55.9% in unfavorable, P = 0.0001), and no grade ≥3 toxicity was observed.
Que et al. in 2016 (52)	26–40 Gy in 3–5 fractions	In this retrospective study of 115 patients with unresectable HCC treated with CyberKnife SBRT, the objective response rate was 88.7% (48.7% CR, 40% PR), median overall survival was 15 months with 1-year OS of 63.5%, 1-year in-field recurrence-free survival was 85.3%, and acute toxicity was mostly transient and tolerable, with Child-Pugh score, PVTT, tumor size, and response identified as independent predictors of OS.
Ze-Tian Shen et al. in 2017 (89)	36–54 Gy in 3–5 fractions	In 28 patients with intrahepatic cholangiocarcinoma treated with CyberKnife SBRT, the disease control rate was 89.3%, median overall survival was 15 months (1-year OS 57.1%, 2-year OS 32.1%), number of lesions, CA19–9 levels, and TNM stage were independent prognostic factors, and no grade ≥3 toxicity was observed.
Zhang et al. in 2018 (90)	35–60 Gy in 3–6 fractions	In 28 patients with small HCC (median size 2.1 cm) treated with CyberKnife SBRT, the overall response rate was 89.3% (60.7% CR), 3-year overall survival was 78.6%, 3-year local control was 89.3%, and no grade ≥3 hepatic toxicity was observed, demonstrating that CyberKnife SBRT is a safe and effective alternative for patient’s ineligible for surgery or ablation.
Ishigaki et al. in 2019 (91)	8–48 Gy in 1–10 fractions	In 13 patients with 60 bone metastases from differentiated thyroid cancer treated with CyberKnife SRT, the disease control rate was 97.5% (2 PR, 37 SD, 1 PD) with a 1-year local control rate of 97.1%, and adverse events were infrequent and mild, demonstrating that CyberKnife SRT is a feasible and effective treatment for DTC bone metastases.
Jing Sun et al. in 2020 (92)	45–54 Gy in 5–10 fractions	In 32 patients with decompensated cirrhosis HCC (Child-Pugh scores 7–10) treated with CyberKnife SBRT, overall survival rates at 1, 2, and 3 years were 84.4%, 61.8%, and 46.0% respectively, local control was 92.9% at all three time points, and liver injury was acceptable, demonstrating that CyberKnife SBRT is an effective option for this challenging patient population.
Acker et al. in 2020 (93)	12–21 Gy in single fraction	In 97 elderly patients (≥65 years, mean age 73.2) with 233 brain metastases treated with CyberKnife SRS (median PTV 1.05 cm³, median dose 19 Gy in single fraction in 92.3%), the 12-month local tumor progression-free interval was 89.0%, 12-month overall survival was 23%, 97.9% maintained stable Karnofsky performance score, only one patient developed Grade 2 neurological deficit, and older age and female sex were predictive of local progression.
Ryuno et al. in 2022 (94)	54–60 Gy in 3 fractions	In 96 patients with stage-I peripheral NSCLC treated with CyberKnife SBRT, the 2-year local control, progression-free survival, and overall survival rates were 97%, 84%, and 90% respectively, with only 1% Grade 3 radiation pneumonitis, although patients with T1c/T2a disease had significantly worse outcomes (90% LC, 65% PFS) compared to T1a/T1b (100% LC, 95% PFS).
Hayashi et al. in 2023 (95)	Peripheral: 52 Gy/4 fx, central: 60 Gy/8–10 fx, median max GTV dose 61.0 Gy	In 73 patients with 112 metastatic lung tumors treated with CyberKnife using a central high-dose technique, the 2-year local control, PFS, and OS rates were 89.1%, 37.1%, and 71.3% respectively, with grade 2 and 3 radiation pneumonitis occurring in only one patient each, both of whom received simultaneous irradiation at 2–3 sites.
Xi Cheng et al. in 2025 (96)	30 to 56 Gy in 3–7 fractions	In 52 HCC patients with adrenal metastases treated with CyberKnife SBRT, the overall response rate was 86.0%, local control rates at 6 months, 1 year, and 2 years were 100%, 81.7%, and 75.8% respectively, median overall survival was 22 months, no grade ≥2 adverse reactions were observed, and intrahepatic tumor control (P = 0.01) and absence of extra-organ metastases (P = 0.001) were independent prognostic factors for improved survival.

Re-irradiation has emerged as an important clinical application for the CyberKnife system, particularly for recurrent or metastatic lesions in previously irradiated sites. The system’s sub-millimeter accuracy, steep dose gradients, real-time tracking, and capability for rigid and deformable image registration make it well-suited for delivering additional radiation while minimizing cumulative dose to OARs (54–58). Several retrospective studies have reported promising outcomes with CyberKnife-based re-irradiation. For example, in a study of 77 patients with 254 locally recurrent brain metastases, CyberKnife SRS achieved local control rates of 92.2%, 73.4%, and 73.4% at 3, 6, and 9 months, respectively, with acceptable toxicity (radionecrosis in only 3 patients). In prostate cancer, a series of 38 patients with local recurrence after prior radiotherapy demonstrated an 86.8% local control rate with generally mild toxicity using CyberKnife radioablation (55). Similar encouraging results have been observed in recurrent chordomas (56) and gliomas (57). These studies highlight the technical advantages of the CyberKnife platform — particularly real-time tracking, rigid and deformable image registration methods, and highly conformal dose delivery — that enable meaningful tumor control in complex re-irradiation scenarios while maintaining generally acceptable toxicity. Nevertheless, important safety concerns remain. The incidence of radiation-induced brain necrosis after re-irradiation with SRS is reported to range from 10–20% (with overall reported rates after SRS varying widely from 0% to 30% in the broader literature), and some cases can be severe (59). Moreover, distinguishing radiation necrosis from true tumor progression on imaging is often challenging, and pathological confirmation is not always available. Therefore, careful patient selection (small target volume, good performance status, and adequate time interval from prior radiation) is critical when considering CyberKnife re-irradiation. Prospective studies with longer follow-up are still needed to more firmly establish the long-term efficacy and safety profile of this approach.

## 7 Advantages, limitations, and future directions

The CyberKnife system, as one of the main commercially available platforms for real-time motion tracking, offers platform-specific advantages for stereotactic radiotherapy. Its ability to perform accurate, real-time respiratory motion tracking distinguishes it from many conventional linac-based systems. Like other modern radiotherapy technologies, it enables non-invasive, hypofractionated treatment delivery (typically 1–5 sessions over one week) on an outpatient basis without anesthesia, allowing most patients to maintain normal daily activities (7, 60). In addition to these general benefits of stereotactic radiotherapy, the CyberKnife provides several platform-specific strengths, including a frameless design that enhances patient comfort, robotic delivery with sub-millimeter precision for both rigid and non-rigid tumors, and real-time image guidance that effectively minimizes radiation exposure to surrounding healthy tissues. These features make the system particularly suitable for delivering highly conformal doses to complex targets and for re-irradiation in carefully selected patients who have previously received high radiation doses to the same region (7, 60).

Despite these advantages, the CyberKnife system has several limitations compared with other stereotactic radiation therapy platforms. One of the main drawbacks is the longer treatment time. For example, in the Linac-based radiotherapy, treatment typically takes 5–10 minutes, whereas CyberKnife sessions often last 15–60 minutes due to robotic arm repositioning and platform-specific workflows (6, 9), even though the new model (M6 or S7) with MLC has reduced treatment time to 10–25 minutes. Other limitations include: 1) the absence of volumetric imaging, as cone-beam CT (CBCT) is not integrated, unlike in many modern Linac systems; 2) the use of higher MUs, which may increase peripheral and leakage radiation doses to the patient; and 3) the limited range of the robotic arm, which can restrict the effective delivery of posterior beams (6, 9, 60, 61). In addition, the CyberKnife system, a well-established platform for stereotactic radiotherapy, lacks HyperArc (HA) capability, a notable limitation. In the authors’ view, these limitations highlight a recurring trade-off in the historical development of CyberKnife: superior tracking and robotic flexibility at the cost of treatment efficiency and some dosimetric conveniences available on conventional platforms. While technological iterations (e.g., MLC and faster models) have narrowed this gap, certain inherent constraints of the robotic design persist. Multiple comparative studies have demonstrated the advantages of HA over CyberKnife across different clinical scenarios (62–65). For example, a study comparing HA and MLC-based CyberKnife for brain metastases in 17 cases showed that HA provided better conformity (0.91 vs. 0.77, *p* < 0.01), lower OAR and normal tissue doses, fewer MUs, and shorter beam-on time (*p* < 0.05). However, gradient indexes were similar, while CyberKnife showed higher homogeneity and target doses (*p* < 0.05), suggesting that HA is more efficient and particularly suitable for larger brain metastases (62). Another study on recurrent nasopharyngeal cancer in 15 patients found that both homogeneous and inhomogeneous HA plans provided similar or better target coverage than CyberKnife while reducing OAR doses by approximately 60%; specifically, homogeneous HA offered better conformity and homogeneity, whereas inhomogeneous HA matched CyberKnife’s heterogeneity with faster dose falloff, concluding that HA is a feasible, efficient, and attractive SBRT option for nasopharyngeal cancer (63). A third study comparing HA, CyberKnife, and conventional VMAT for a single large glioblastoma multiforme in 16 patients demonstrated that HA plans achieved higher conformity (CI 0.94 ± 0.03) and lower gradient index (2.57 ± 0.53), resulting in lower OAR and normal brain doses, while CyberKnife produced higher homogeneity and intratumoral doses; notably, HA saved approximately 37 minutes of delivery time compared to CyberKnife, leading to the conclusion that HA offers excellent dosimetric quality and efficiency for single large tumors, with tumor size and location influencing treatment selection (64). Although these studies consistently favor HyperArc in terms of treatment efficiency and OAR sparing for certain intracranial targets, CyberKnife often maintains competitive or superior target homogeneity and remains preferable in cases requiring real-time tracking or irregular target motion. This comparison underscores that no single platform is universally superior; rather, technology selection should be guided by tumor location, size, and the need for continuous motion management — reflecting the ongoing diversification of stereotactic radiotherapy platforms.

It is also important to note that implementing CyberKnife presents several practical challenges. Since SRS and SBRT have only been widely adopted in the past decade, many medical physicists still lack sufficient experience in small-field dosimetry, an essential aspect when working with robotic-based systems. One key implementation difference between the CyberKnife system and conventional Linac-based SRS is the additional mechanical DQA required for the robotic arm used in CyberKnife, compared with the standard gantry mechanics of a conventional Linac. Another challenge is limited access to CyberKnife centers and a shortage of experienced mentors for training and guidance (9, 60).

Deep learning (DL) has become increasingly important in IGRT, offering advanced dose-prediction and beam-modeling capabilities (66–68). In CyberKnife applications, Miao et al. developed a deep CNN-based method for predicting brain SRS doses (67). Their model achieved high gamma passing rates (96.7% for the body, 98.3% for the PTV, and 100% for the miniature OARs) and closely matched the clinical plan’s DVHs, with target dose differences generally below 1.0 Gy (~3%). Results from 14 brain cases demonstrate accurate 3D dose prediction for homogeneous brain tissue, suggesting that DL can accelerate CyberKnife treatment planning, improve efficiency, and optimize clinical workflows. However, larger studies across multiple cancer sites are needed for validation. The same group developed a 3D U-Net model to predict MC–based 3D dose distributions for CyberKnife (66). The anatomy-and-beam (AB) model outperformed the Mask model, showing 20-40% higher dice similarity coefficient (DSC) in some dose regions, >90% improvement in small voxels, and gamma passing rates of ~99% for PTV and >95% for OARs (3 mm/3%). The AB model closely matched clinical DVHs, with an average organ dose error of 1.65 ± 0.69%. This approach enables rapid, accurate prediction and dose optimization for CyberKnife treatment planning in lung cancer. However, it is important to note that none of these deep learning methods have yet been implemented in routine clinical practice for CyberKnife. Future clinical implementation will require further validation, regulatory approval, and integration into commercial treatment planning systems.

## 8 Most recent advances in the CyberKnife system

The CyberKnife system has undergone continuous evolution since its introduction, with the M6 and S7 models representing the most significant advancements to date. These latest generations incorporate several key technological improvements to enhance treatment efficiency, beam delivery flexibility, and patient safety.

Key hardware and design upgrades in the M6 and S7 systems include a fully digital platform, an upgraded KUKA KR300 R2500 Ultra robotic arm, and a redesigned room layout with the robot positioned at the head of the treatment table. These changes expand the treatment workspace and improve posterior oblique beam delivery (up to approximately 20°). The beam delivery system now supports a maximum dose rate of 1000 MU/min. Additionally, the redesigned architecture achieves a more balanced payload distribution across the 3D workspace (50%–50% left–right symmetry compared to the previous 64%–36% imbalance in earlier models), resulting in greater treatment flexibility and efficiency (6, 7). These hardware improvements reflect the ongoing maturation of the CyberKnife platform over its three-decade history, evolving from a highly specialized but somewhat limited robotic system toward greater clinical practicality and workflow efficiency. Nevertheless, even with these upgrades, the fundamental robotic architecture still imposes certain mechanical constraints — particularly regarding full 360° workspace utilization and unrestricted posterior beam delivery — although the new design has notably improved the ability to deliver beams from below the patient.

Beam-shaping capabilities have been significantly enhanced with the introduction of the InCise™ 2 MLC alongside the existing fixed cones and Iris™ variable collimator. The InCise™ 2 MLC offers larger field sizes, improved dose conformity, steeper dose gradients, and notably reduced treatment time and MU requirements. Clinical studies have shown that MLC-based plans can reduce treatment time by 35–50% and MUs by 35–66%, while also decreasing the number of beam nodes by 4–13% compared to Iris or fixed collimators (11, 41, 69). In a study of 15 patients with benign intracranial tumors, MLC plans demonstrated better dose fall-off and conformity, along with a lower estimated risk of secondary malignancy compared to Iris collimators (70). Radiation leakage has also been substantially reduced. While earlier G3 and G4 systems exhibited leakage of approximately 0.5% of the primary beam, the S7 model achieves leakage below 0.05% in all planes, well within IEC 60601-2–1 limits. This improvement enhances patient safety and allows for higher treatment throughput with reduced shielding requirements (35, 71). The integration of InCise™ 2 MLC represents a major milestone in CyberKnife’s technical evolution, substantially addressing previous criticisms regarding long treatment times and high monitor units. These advances have narrowed the efficiency gap with linac-based systems while preserving the platform’s core strengths in conformity and steep dose gradients. However, in the authors’ view, the single-layer InCise MLC still lags behind the double-layer MLC (DL-MLC) commonly used in modern linear accelerators, which employ stacked, staggered, and often rotating leaf banks to achieve even lower radiation leakage and sharper penumbra.

Significant progress has also been made in target tracking. In addition to the five commonly used methods (6D skull tracking, fiducial tracking, Synchrony respiratory tracking, Xsight spine tracking, and Xsight lung tracking) (41, 72, 73), newer techniques such as Xsight spine prone tracking and LOT have expanded clinical applicability. Xsight spine-prone tracking enables fiducial-free treatment of lower lumbosacral lesions in the prone position using combined respiratory and skeletal tracking (74, 75). The LOT technique allows real-time lung tumor tracking using 0-, 1-, or 2-view imaging without implanted fiducials, minimizing invasiveness while maintaining high accuracy. The latest Synchrony system further integrates AI-based predictive algorithms, enabling real-time motion compensation for respiratory-moving targets. When combined with the VOLO optimizer, these advancements have reduced typical treatment session times to as little as 15 minutes for many hypofractionated SRS and SBRT cases (7).

## 9 Discussion

Over the past three decades, the CyberKnife robotic radiosurgery system has played a pioneering role in the development of SRS and SBRT. Its distinctive integration of a six-degree-of-freedom (6DOF) robotic arm, real-time image guidance, and advanced respiratory motion tracking (particularly the Synchrony system) has enabled sub-millimeter targeting accuracy for both static and moving targets, significantly expanding the treatable indications in anatomically challenging locations such as the brain, spine, lung, liver, pancreas, and prostate. The system also offers clear technical advantages in delivering highly conformal doses with steep dose gradients and in managing intrafraction motion without the need for breath-hold or gating.

Nevertheless, accumulating evidence indicates that CyberKnife no longer maintains exclusive superiority across all clinical scenarios. Comparative dosimetric and clinical studies have shown that modern C-arm linac-based SBRT platforms, equipped with advanced IGRT, non-coplanar VMAT, and HyperArc, can achieve target coverage and organ-at-risk sparing comparable to those achieved with other platforms. In particular, linac-based techniques frequently demonstrate substantial advantages in treatment efficiency, including significantly shorter delivery times, lower integral doses to normal tissue, and higher patient throughput. For instance, HyperArc has been shown to provide superior conformity and normal brain sparing for multiple brain metastases, while also reducing beam-on time by a considerable amount compared with CyberKnife (48–51, 62–64).

The results of **Table 2** summarize clinical outcomes and treatment parameters for CyberKnife SBRT across tumor sites. However, C-arm Linac-based SBRT (e.g., VMAT) provides comparable local control and survival rates for tumors, often exceeding 90% for early-stage disease. For example, comparative studies in early-stage NSCLC have shown largely comparable outcomes between CyberKnife and linac-based SBRT. A prospective multicenter study of 106 patients with peripheral lesions found no significant differences in 2-year local control (97% with Linac vs. 100% with CyberKnife), progression-free survival, overall survival, or toxicity (50). Similarly, a large prospective study of 401 medically inoperable patients treated with identical dose regimens reported excellent 2-year local control (94%) and low toxicity for both platforms (76). In contrast, one study of 422 stage I patients suggested a potential dosimetric advantage for CyberKnife, demonstrating significantly higher mean dose to the peri-PTV shell (38.1 Gy vs. 22.8 Gy), which was associated with improved 2-year local control (96% vs. 88%) and better distant metastasis-free survival (46). Collectively, these findings indicate that while CyberKnife may provide a steeper peripheral dose gradient in some cases, both modalities yield high local control and acceptable toxicity when appropriate doses are delivered, supporting platform selection based on institutional expertise and practical considerations rather than clear clinical superiority.

Key limitations of the CyberKnife system include relatively long treatment session time (even after MLC and S7 upgrades), the lack of volumetric cone-beam CT (CBCT) imaging, higher monitor units in certain plans, high capital and maintenance costs, and restricted posterior beam access due to the robotic arm’s workspace limitations and patient positioning. Consequently, CyberKnife is not suitable for all patients. Its effectiveness is reduced for large tumors (>5–6 cm), certain tumor types in proximity to critical organs, patients with widespread metastatic disease, or those who have already received near-maximum radiation doses to surrounding tissues. The system is most effective for small to medium-sized lesions, particularly early-stage localized lung cancer, brain metastases (single or multiple), re-irradiation of recurrent lesions in previously irradiated fields, and spinal tumors involving no more than three vertebral levels, where excellent spinal cord sparing can be achieved (41–43, 77, 78).

Several limitations of this review should be acknowledged, including the heterogeneity of the included studies, the predominance of retrospective and dosimetric data, and the generally low-to-moderate quality of the available evidence. Rapid technological advances in both robotic and linac-based systems further limit the generalizability of older findings. To address these limitations, future research should prioritize head-to-head randomized controlled trials comparing CyberKnife with other SBRT platforms (e.g., C-arm linac-based, MR-guided systems) for key clinical indications, as well as robust cost-effectiveness analyses to determine the long-term value of robotic stereotactic radiotherapy in routine clinical practice.

## 10 Summary and conclusions

In summary, the CyberKnife system is particularly valuable for tumors near critical structures, small to medium-sized lesions, and targets affected by respiratory motion. Although its treatment cost may be higher, its technical capabilities offer distinct advantages for complex scenarios, such as treating tumors in challenging locations or managing respiratory motion, ultimately leading to improved clinical outcomes that justify its use in select cases.

Nevertheless, rapid advances in linac-based SBRT, especially with non-coplanar VMAT, HyperArc, and improved IGRT, have narrowed the performance gap considerably. While CyberKnife retains distinct advantages in continuous real-time tracking and highly conformal dose delivery for selected challenging targets, modern linac platforms often provide comparable clinical outcomes with superior treatment efficiency, lower integral dose, and greater accessibility. Additionally, CyberKnife should not be considered a universal solution but rather a complementary technology best reserved for cases where its unique technical features — particularly real-time respiratory tracking and exceptional dose conformity near critical structures — offer clear clinical benefit. Platform selection must be individualized based on tumor size, location, motion characteristics, proximity to OARs, and institutional resources.

Future well-designed prospective comparative trials, together with continued integration of artificial intelligence into planning and motion management, will be essential to further define the precise role of robotic radiosurgery in the era of modern precision radiotherapy.

## References

[1] Miszczyk J Rawojć K Panek A Borkowska A Prasanna P Ahmed M . Do protons and X-rays induce cell-killing in human peripheral blood lymphocytes by different mechanisms? Clin Trans Radiat Oncol. (2018) 9:23–9. doi: 10.1016/j.ctro.2018.01.004 29594247 PMC5862687

[2] Dörr W Gabryś D . The principles and practice of re-irradiation in clinical oncology: an overview. Clin Oncol. (2018) 30:67–72. doi: 10.1016/j.clon.2017.11.014 29233574

[3] Grégoire V Guckenberger M Haustermans K Lagendijk JJ Ménard C Pötter R . Image guidance in radiation therapy for better cure of cancer. Mol Oncol. (2020) 14:1470–91. doi: 10.1002/1878-0261.12751 PMC7332209 32536001

[4] Leksell L . The stereotactic method and radiosurgery of the brain. Acta Chir Scand. (1951) 102:316–9. doi: 10.1097/00006123-198902000-00026 14914373

[5] Schmitt D Blanck O Gauer T Fix MK Brunner TB Fleckenstein J . Technological quality requirements for stereotactic radiotherapy: Expert review group consensus from the DGMP Working Group for Physics and Technology in Stereotactic Radiotherapy. Strahlenther Onkol. (2020) 196:421–43. doi: 10.1007/s00066-020-01583-2 32211939 PMC7182540

[6] Kilby W Dooley J Kuduvalli G Sayeh S Maurer C . The CyberKnife® robotic radiosurgery system in 2010. Technol Cancer Res Treat. (2010) 9:433–52. doi: 10.1177/153303461000900502 20815415

[7] Kilby W Naylor M Dooley JR Maurer CR Sayeh S . A technical overview of the CyberKnife system. In: Handbook of robotic and image-guided surgery. Elsevier (2020). p. 15–38.

[8] Adler JR Chang SD Murphy MJ Doty J Geis P Hancock SL . The Cyberknife: a frameless robotic system for radiosurgery. Stereotact Funct Neurosurg. (1997) 69:124–8. doi: 10.1159/000099863 9711744

[9] Pollom E Wang L Gibbs IC Soltys SG . CyberKnife robotic stereotactic radiosurgery. In: Stereotactic radiosurgery and stereotactic body radiation therapy. Springer (2019). p. 67–76.

[10] Murphy MJ . An automatic six‐degree‐of‐freedom image registration algorithm for image‐guided frameless stereotaxic radiosurgery. Med Phys. (1997) 24:857–66. doi: 10.1118/1.598005 9198019

[11] Moutsatsos A Pantelis E . The CyberKnife robotic radiosurgery system. In: CyberKnife neuroRadiosurgery. Springer (2020). p. 31–43.

[12] Kawata K Kamomae T Oguchi H Kawabata F Okudaira K Kawamura M . Evaluation of newly implemented dose calculation algorithms for multileaf collimator‐based CyberKnife tumor‐tracking radiotherapy. Med Phys. (2020) 47:1391–403. doi: 10.1002/mp.14013 31913508

[13] Li J Zhang X Pan Y Zhuang H Yang R . Comparison of ray tracing and monte carlo calculation algorithms for spine lesions treated with cyberKnife. Front Oncol. (2022) 12:898175. doi: 10.21203/rs.3.rs-280719/v1 35600341 PMC9116717

[14] Mukwada G Skorska M Rowshanfarzad P Ebert MA . Comparison of the accuracy of Monte Carlo and Ray Tracing dose calculation algorithms for multiple target brain treatments on CyberKnife. Phys Eng Sci Med. (2023) 46:1477–87. doi: 10.1007/s13246-023-01312-w 37552365

[15] Ge X Yang M Li T Liu T Gao X Qiu Q . Comparative analysis of dose calculation algorithms for CyberKnife-based stereotactic radiotherapy in lung cancer. Front Oncol. (2023) 13:1215976. doi: 10.3389/fonc.2023.1215976 37849803 PMC10577380

[16] Depuydt T Poels K Verellen D Engels B Collen C Buleteanu M . Treating patients with real-time tumor tracking using the Vero gimbaled linac system: implementation and first review. Radiother Oncol. (2014) 112:343–51. doi: 10.1016/j.radonc.2014.05.017 25049177

[17] Depuydt T Poels K Verellen D Engels B Collen C Haverbeke C . Initial assessment of tumor tracking with a gimbaled linac system in clinical circumstances: a patient simulation study. Radiother Oncol. (2013) 106:236–40. doi: 10.1016/j.radonc.2012.12.015 23398905

[18] Poels K Dhont J Verellen D Blanck O Ernst F Vandemeulebroucke J . A comparison of two clinical correlation models used for real-time tumor tracking of semi-periodic motion: a focus on geometrical accuracy in lung and liver cancer patients. Radiother Oncol. (2015) 115:419–24. doi: 10.1016/j.radonc.2015.05.004 25981054

[19] Miandoab PS Worm E Hansen R Weber B Høyer M Saramad S . Accuracy of four models and update strategies to estimate liver tumor motion from external respiratory motion. Front Oncol. (2024) 14:1470650. doi: 10.3389/fonc.2024.1470650 39381048 PMC11458717

[20] Ge J Santanam L Yang D Parikh PJ . Accuracy and consistency of respiratory gating in abdominal cancer patients. Int J Radiat Oncol Biol Phys. (2013) 85:854–61. doi: 10.1016/j.ijrobp.2012.05.006 22717241

[21] Hickling SV Veres AJ Moseley DJ Grams MP . Implementation of free breathing respiratory amplitude‐gated treatments. J Appl Clin Med Phys. (2021) 22:119–29. doi: 10.1002/acm2.13253 33982875 PMC8200514

[22] Keall PJ Nguyen DT O'Brien R Caillet V Hewson E Poulsen PR . The first clinical implementation of real-time image-guided adaptive radiotherapy using a standard linear accelerator. Radiother Oncol. (2018) 127:6–11. doi: 10.1016/j.radonc.2018.01.001 29428258

[23] Bertholet J Worm ES Fledelius W Høyer M Poulsen PR . Time-resolved intrafraction target translations and rotations during stereotactic liver radiation therapy: implications for marker-based localization accuracy. Int J Radiat Oncol Biol Phys. (2016) 95:802–9. doi: 10.1016/j.ijrobp.2016.01.033 27020108

[24] Rankine L Wan H Parikh P Maughan N Poulsen P DeWees T . Cone-beam computed tomography internal motion tracking should be used to validate 4-dimensional computed tomography for abdominal radiation therapy patients. Int J Radiat Oncol Biol Phys. (2016) 95:818–26. doi: 10.1016/j.ijrobp.2016.01.047 27020102

[25] Cho B Poulsen PR Keall PJ . Real-time tumor tracking using sequential kV imaging combined with respiratory monitoring: a general framework applicable to commonly used IGRT systems. Phys Med Biol. (2010) 55:3299. doi: 10.1088/0031-9155/55/12/003 20484777 PMC2974817

[26] Worm ES Thomsen JB Johansen JG Poulsen PR . A simple method to measure the gating latencies in photon and proton based radiotherapy using a scintillating crystal. Med Phys. (2023) 50:3289–98. doi: 10.1002/mp.16418 37075173

[27] Samadi Miandoab P Liu Y Shang X Lv T Xu H Zhang G . Feasibility study of using CNN‐GRU‐Dense model for real‐time liver tumor tracking during radiotherapy. Med Phys. (2025) 52:e70014. doi: 10.1002/mp.70014 40975844

[28] Miandoab PS Setayeshi S Blanck O Saramad S . Feasibility study of using next-generation reservoir computing (NG-RC) model to estimate liver tumor motion from external breathing signals. Med Phys. (2025) 52:1416–29. doi: 10.1002/mp.17595 39714092

[29] Samadi Miandoab P Saramad S Setayeshi S . Respiratory motion prediction based on deep artificial neural networks in CyberKnife system: A comparative study. J Appl Clin Med Phys. (2023) 24:e13854. doi: 10.1002/acm2.13854 36457192 PMC10018664

[30] Zhang J Wang L Li X Huang M Xu B . Quantification of intrafraction and interfraction tumor motion amplitude and prediction error for different liver tumor trajectories in Cyberknife synchrony tracking. Int J Radiat Oncol Biol Phys. (2021) 109:1588–605. doi: 10.1016/j.ijrobp.2020.11.036 33227440

[31] Remy C Ahumada D Labine A Côté J-C Lachaine M Bouchard H . Potential of a probabilistic framework for target prediction from surrogate respiratory motion during lung radiotherapy. Phys Med Biol. (2021) 66:105002. doi: 10.1088/1361-6560/abf1b8 33761479

[32] Samadi Miandoab P Saramad S Setayeshi S Blanck O . A retrospective multi‐center feasibility study of a new PTV margin estimation approach for moving targets using CyberKnife log files. J Appl Clin Med Phys. (2023) 24:e13975. doi: 10.1002/acm2.13975 37004149 PMC10338771

[33] Samadi Miandoab P Saramad S Setayeshi S . Target margin design through analyzing a large cohort of clinical log data in the cyberknife system. J Appl Clin Med Phys. (2022) 23:e13476. doi: 10.1002/acm2.13476 35044071 PMC8906228

[34] Chan M Grehn M Cremers F Siebert F-A Wurster S Huttenlocher S . Dosimetric implications of residual tracking errors during robotic SBRT of liver metastases. Int J Radiat Oncol Biol Phys. (2017) 97:839–48. doi: 10.1016/j.ijrobp.2016.11.041 28244421

[35] Wang L Descovich M Wilcox EE Yang J Cohen AB Fuerweger C . AAPM task group report 135. B: Quality assurance for robotic radiosurgery. Med Phys. (2025) 52:45–76. doi: 10.1007/978-3-540-85516-3_656 39453412 PMC11700000

[36] Nakayama M Nishimura H Mayahara H Nakamura M Uehara K Tsudou S . Clinical log data analysis for assessing the accuracy of the CyberKnife fiducial-free lung tumor tracking system. Pract Radiat Oncol. (2018) 8:e63–70. doi: 10.1016/j.prro.2017.10.014 29329997

[37] Pantelis E Moutsatsos A Antypas C Zoros E Pantelakos P Lekas L . On the total system error of a robotic radiosurgery system: phantom measurements, clinical evaluation and long-term analysis. Phys Med Biol. (2018) 63:165015. doi: 10.1088/1361-6560/aad516 30033940

[38] Oliver PA Yewondwossen M Summers C Shaw C Cwajna S Syme A . Influence of intra-and interfraction motion on planning target volume margin in liver stereotactic body radiation therapy using breath hold. Adv Radiat Oncol. (2021) 6:100610. doi: 10.1016/j.adro.2020.10.023 33490733 PMC7809510

[39] Jasper K Liu B Olson R Matthews Q . Evidence-based planning target volume margin reduction for modern lung stereotactic ablative radiation therapy using deformable registration. Adv Radiat Oncol. (2021) 6:100750. doi: 10.1016/j.adro.2021.100750 34401609 PMC8349747

[40] Cheng Y Lin Y Long Y Du L Chen R Hu T . Is the CyberKnife® radiosurgery system effective and safe for patients? An umbrella review of the evidence. Future Oncol. (2022) 18:1777–91. doi: 10.2217/fon-2021-0844 35137603

[41] Ding C Saw CB Timmerman RD . Cyberknife stereotactic radiosurgery and radiation therapy treatment planning system. Med Dosim. (2018) 43:129–40. doi: 10.1016/j.meddos.2018.02.006 29605528

[42] Donati CM Medici F Zamfir AA Galietta E Cammelli S Buwenge M . CyberKnife in pediatric oncology: a narrative review of treatment approaches and outcomes. Curr Oncol. (2025) 32:76. doi: 10.3390/curroncol32020076 39996876 PMC11854067

[43] Grishchuk D Dimitriadis A Sahgal A De Salles A Fariselli L Kotecha R . ISRS technical guidelines for stereotactic radiosurgery: Treatment of small brain metastases (≤ 1 cm in diameter). Pract Radiat Oncol. (2023) 13:183–94. doi: 10.1016/j.prro.2022.10.013 36435388

[44] Temming S Kocher M Stoelben E Hagmeyer L Chang D-H Frank K . Risk-adapted robotic stereotactic body radiation therapy for inoperable early-stage non-small-cell lung cancer. Strahlenther Onkol. (2018) 194:91–7. doi: 10.1007/s00066-017-1194-x 28812120

[45] Ihnát P Skácelíková E Tesař M Penka I . Stereotactic body radiotherapy using the CyberKnife® system in the treatment of patients with liver metastases: state of the art. Onco Targets Ther. (2018) 11:4685–91. doi: 10.2147/OTT.S165878 PMC6091471 30127616

[46] Diamant A Heng VJ Chatterjee A Faria S Bahig H Filion E . Comparing local control and distant metastasis in NSCLC patients between CyberKnife and conventional SBRT. Radiother Oncol. (2020) 144:201–8. doi: 10.1016/j.radonc.2020.01.017 32044418

[47] Ratnakumaran R Sasitharan A Khan A Mayet H Mohajer J Hinder V . Dosimetric comparison of CyberKnife and conventional LINAC prostate stereotactic body radiation therapy plans: analysis of the PACE-B study. Int J Radiat Oncol Biol Phys. (2025) 125(1):250–6. doi: 10.1016/j.ijrobp.2025.01.014 39862896

[48] Tawfik ZA Farid M-A El Shahat KM Hussein AA Eldib AA Al Etreby M . A dosimetric study comparing Cyberknife and LINAC-based stereotactic radiotherapy or radiosurgery treatments. J Radiat Res Appl Sci. (2024) 17:100781. doi: 10.1016/j.jrras.2023.100781 38826717

[49] Baydoun A Vapiwala N Ponsky LE Awan M Kassaee A Sutton D . Comparative analysis for renal stereotactic body radiotherapy using Cyberknife, VMAT and proton therapy based treatment planning. J Appl Clin Med Phys. (2018) 19:125–30. doi: 10.1002/acm2.12308 29542260 PMC5978559

[50] Claude L Morelle M Mahé M-A Pasquier D Boisselier P Bondiau PY . A comparison of two modalities of stereotactic body radiation therapy for peripheral early-stage non-small cell lung cancer: results of a prospective French study. Br J Radiol. (2020) 93:20200256. doi: 10.1259/bjr.20200256 32970478 PMC7716015

[51] Zhang Y Chiu T Dubas J Tian Z Lee P Gu X . Benchmarking techniques for stereotactic body radiotherapy for early-stage glottic laryngeal cancer: LINAC-based non-coplanar VMAT vs. Cyberknife planning. Radiat Oncol. (2019) 14:193. doi: 10.1186/s13014-019-1404-z 31684993 PMC6829943

[52] Que J Kuo H-T Lin L-C Lin K-L Lin C-H Lin Y-W . Clinical outcomes and prognostic factors of cyberknife stereotactic body radiation therapy for unresectable hepatocellular carcinoma. BMC Cancer. (2016) 16:1–10. doi: 10.1186/s12885-016-2512-x 27405814 PMC4941022

[53] Ji X Zhao Y He C Han S Zhu X Shen Z . Clinical effects of stereotactic body radiation therapy targeting the primary tumor of liver-only oligometastatic pancreatic cancer. Front Oncol. (2021) 11:659987. doi: 10.3389/fonc.2021.659987 34123818 PMC8190391

[54] Berber T Raturi V Aksaray F Hojo H Fujisawa T Ohyoshi H . Clinical outcome after CyberKnife® radiosurgery re-irradiation for recurrent brain metastases. Cancer/Radiothérapie. (2021) 25:457–62. doi: 10.1016/j.canrad.2021.02.003 33752961

[55] Miszczyk L Stąpór-Fudzińska M Miszczyk M Maciejewski B Tukiendorf A . Salvage cyberknife-based reirradiation of patients with recurrent prostate cancer: the single-center experience. Technol Cancer Res Treat. (2018) 17:1533033818785496. doi: 10.1177/1533033818785496 29983098 PMC6048607

[56] Berber T Numanoğlu Ç Uysal E Dinçer S Yıldırım BA . Results of salvage treatment with CyberKnife® fractioned radiosurgery in recurrent large chordoma. Eur Spine J. (2023) 32:244–53. doi: 10.1007/s00586-022-07399-1 36180739

[57] Adachi K Hayashi K Kagawa N Kinoshita M Sumida I Akino Y . Feasibility of salvage re-irradiation with stereotactic radiotherapy for recurrent glioma using CyberKnife. Anticancer Res. (2019) 39:2935–40. doi: 10.21873/anticanres.13423 31177132

[58] Zhou H Wu T Zhu X Li Y . Re-irradiation of multiple brain metastases using CyberKnife stereotactic radiotherapy: case report. Medicine. (2021) 100:e27543. doi: 10.1097/md.0000000000027543 34731155 PMC8519193

[59] Mayo ZS Billena C Suh JH Lo SS Chao ST . The dilemma of radiation necrosis from diagnosis to treatment in the management of brain metastases. Neuro Oncol. (2024) 26:S56–65. doi: 10.1093/neuonc/noad188 38437665 PMC10911797

[60] Eshleman JS Berkenstock KG Singapuri KP Fuller C . The CyberKnife® M6™ radiosurgery system. J Lanc Gen Hosp. (2013) 8:44–9. Available online at: https://www.jlgh.org/JLGH/media/Journal-LGH-Media-Library/Past%20Issues/Volume%208%20-%20Issue%202/Eshleman8_2.pdf

[61] Fürweger C Drexler C Muacevic A Wowra B de Klerck EC Hoogeman MS . CyberKnife robotic spinal radiosurgery in prone position: dosimetric advantage due to posterior radiation access? J Appl Clin Med Phys. (2014) 15:11–21. doi: 10.1120/jacmp.v15i4.4427 PMC5875502 25207392

[62] Zhu L Dong S Sun L Xiao Y Zhong Y Pan M . Dosimetric comparison of HyperArc and InCise MLC‐based CyberKnife plans in treating single and multiple brain metastases. J Appl Clin Med Phys. (2024) 25:e14404. doi: 10.1002/acm2.14404 38803034 PMC11302820

[63] Ho H-W Yang C-C Lin H-M Chen H-Y Huang C-C Wang S-C . The feasibility and efficacy of new SBRT technique HyperArc for recurrent nasopharyngeal carcinoma: noncoplanar cone-based robotic system vs. noncoplanar high-definition MLC based Linac system. Med Dosim. (2021) 46:164–70. doi: 10.1016/j.meddos.2020.10.007 33208290

[64] Pan M Xu W Sun L Wang C Dong S Guan Y . Dosimetric quality of HyperArc in boost radiotherapy for single glioblastoma: comparison with CyberKnife and manual VMAT. Radiat Oncol. (2023) 18:8. doi: 10.1186/s13014-022-02150-y 36627633 PMC9832781

[65] Kadoya N Abe Y Kajikawa T Ito K Yamamoto T Umezawa R . Automated noncoplanar treatment planning strategy in stereotactic radiosurgery of multiple cranial metastases: HyperArc and CyberKnife dose distributions. Med Dosim. (2019) 44:394–400. doi: 10.1016/j.meddos.2019.02.004 30827765

[66] Miao Y Li J Ge R Xie C Liu Y Zhang G . Dose prediction of CyberKnife Monte Carlo plan for lung cancer patients based on deep learning: robust learning of variable beam configurations. Radiat Oncol. (2024) 19:170. doi: 10.1186/s13014-024-02531-5 39587661 PMC11587619

[67] Miao Y Ge R Xie C Dai X Liu Y Qu B . Three-dimensional dose prediction based on deep convolutional neural networks for brain cancer in CyberKnife: accurate beam modelling of homogeneous tissue. BJR| Open. (2024) 6:tzae023. doi: 10.1093/bjro/tzae023 39220325 PMC11364489

[68] Jiao S Xu H Luo J Lei L Zhou P . Rapid dose prediction for lung CyberKnife radiotherapy plans utilizing a deep learning approach by incorporating dosimetric features delivered by noncoplanar beams. BioMed Phys Eng Express. (2025) 11:037002. doi: 10.1088/2057-1976/adc697 40153867

[69] McGuinness CM Gottschalk AR Lessard E Nakamura JL Pinnaduwage D Pouliot J . Investigating the clinical advantages of a robotic linac equipped with a multileaf collimator in the treatment of brain and prostate cancer patients. J Appl Clin Med Phys. (2015) 16:284–95. doi: 10.1120/jacmp.v16i5.5502 26699309 PMC5690182

[70] Chao P-J Lee T-F . A mini review of plan quality and secondary cancer risk in CyberKnife M6 radiosurgery for benign intracranial tumors. Front Oncol. (2024) 14:1453256. doi: 10.3389/fonc.2024.1453256 39175469 PMC11339786

[71] Singh S Bhushan M Singh PK Sahoo D Sen S Dipesh . Quantification of head leakage radiation in CyberKnife robotic radiosurgery systems using a multimodal approach. Sci Rep. (2025) 15:34913. doi: 10.1038/s41598-025-18689-1 41057492 PMC12504714

[72] Klein TJ Gill S Ebert MA Grogan G Smith W Alkhatib Z . CyberKnife Xsight versus fiducial-based target-tracking: a novel 3D dosimetric comparison in a dynamic phantom. Radiat Oncol. (2022) 17:154. doi: 10.21203/rs.3.rs-1542801/v1 36076249 PMC9461108

[73] Zhang J Wang L Xie C Yang Z Xu B Li X . Novel utilization and quantification of Xsight diaphragm tracking for respiratory motion compensation in Cyberknife Synchrony treatment of liver tumors. J Appl Clin Med Phys. (2024) 25:e14341. doi: 10.1002/acm2.14341 38622894 PMC11244677

[74] Li J Kong X Cheng C Wang G Zhuang H Yang R . Comparison between supine and prone patient setup for lumbosacral spinal stereotactic body radiosurgery with CyberKnife. Front Oncol. (2023) 13:959447. doi: 10.3389/fonc.2023.959447 37077832 PMC10106579

[75] Mizonobe K Akasaka H Uehara K Oki Y Nakayama M Tamura S . Respiratory motion tracking of spine stereotactic radiotherapy in prone position. J Appl Clin Med Phys. (2023) 24:e13910. doi: 10.1002/acm2.13910 36650923 PMC10161010

[76] Jánváry ZL Bajcsay A Stelczer G Kontra G Pócza T Gerdán M . Long-term clinical results of early-stage lung cancer patients treated with risk-adapted stereotactic body radiotherapy using LINAC or CyberKnife: A single-institution analysis of more than 400 cases. Strahlenther Onkol. (2025) 201:1208–18. doi: 10.1007/s00066-025-02455-3 PMC12546400 40853545

[77] Dupic G Huertas A Nassef M Cosset J-M . Place of Linacs in extracranial stereotactic radiotherapy: Are they now equivalent to Cyberknife®? Bull Cancer. (2022) 109:338–45. doi: 10.1016/j.bulcan.2021.10.008 35090720

[78] Smith JA Jivraj J Wong R Yang V . 30 years of neurosurgical robots: review and trends for manipulators and associated navigational systems. Ann BioMed Eng. (2016) 44:836–46. doi: 10.1007/s10439-015-1475-4 26467553

[79] Yu C Main W Taylor D Kuduvalli G Apuzzo ML Adler JR . An anthropomorphic phantom study of the accuracy of Cyberknife spinal radiosurgery. Neurosurgery. (2004) 55:1138–49. doi: 10.1227/01.neu.0000141080.54647.11 15509320

[80] Antypas C Pantelis E . Performance evaluation of a CyberKnife® G4 image-guided robotic stereotactic radiosurgery system. Phys Med Biol. (2008) 53:4697. doi: 10.1088/0031-9155/53/17/016 18695294

[81] Nano TF Capaldi DP Yeung T Chuang CF Wang L Descovich M . Performance of CyberKnife® tracking using low‐dose CT and kV imaging. Med Phys. (2020) 47:6163–70. doi: 10.1002/mp.14537 33064863

[82] Jung J Song SY Yoon SM Kwak J Yoon K Choi W . Verification of accuracy of CyberKnife tumor-tracking radiation therapy using patient-specific lung phantoms. Int J Radiat Oncol Biol Phys. (2015) 92:745–53. doi: 10.1016/j.ijrobp.2015.02.055 25936598

[83] Winter JD Wong R Swaminath A Chow T . Accuracy of robotic radiosurgical liver treatment throughout the respiratory cycle. Int J Radiat Oncol Biol Phys. (2015) 93:916–24. doi: 10.1016/j.ijrobp.2015.08.031 26530762

[84] Yang J Lamond JP Feng J Wu X Lanciano R Brady LW . CyberKnife system. In: Stereotactic body radiation therapy. Springer (2012). p. 37–52.

[85] Okamoto H Hamada M Sakamoto E Wakita A Nakamura S Kato T . Log-file analysis of accuracy of beam localization for brain tumor treatment by CyberKnife. Pract Radiat Oncol. (2016) 6:e361–7. doi: 10.1016/j.prro.2016.01.008 27053497

[86] Chang U-K Youn SM Park SQ Rhee CH . Clinical results of Cyberknife® radiosurgery for spinal metastases. J Korean Neurosurgical Soc. (2009) 46:538. doi: 10.3340/jkns.2009.46.6.538 20062569 PMC2803269

[87] Que JY Lin L-C Lin K-L Lin C-H Lin Y-W Yang C-C . The efficacy of stereotactic body radiation therapy on huge hepatocellular carcinoma unsuitable for other local modalities. Radiat Oncol. (2014) 9:120. doi: 10.1186/1748-717x-9-120 24885086 PMC4055213

[88] Yuan Z-Y Meng M-B Liu C-L Wang H-H Jiang C Song Y-C . Stereotactic body radiation therapy using the CyberKnife® system for patients with liver metastases. Onco Targets Ther. (2014) 7:915–23. doi: 10.2147/ott.s58409 24959080 PMC4061159

[89] Shen Z-T Zhou H Li A-M Li B Shen J-S Zhu X-X . Clinical outcomes and prognostic factors of stereotactic body radiation therapy for intrahepatic cholangiocarcinoma. Oncotarget. (2017) 8:93541. doi: 10.18632/oncotarget.19972 29212171 PMC5706817

[90] Zhang T Sun J He W Li H Piao J Xu H . Stereotactic body radiation therapy as an effective and safe treatment for small hepatocellular carcinoma. BMC Cancer. (2018) 18:1–8. doi: 10.1186/s12885-018-4359-9 29678159 PMC5910595

[91] Ishigaki T Uruno T Sugino K Masaki C Akaishi J Hames KY . Stereotactic radiotherapy using the CyberKnife is effective for local control of bone metastases from differentiated thyroid cancer. J Radiat Res. (2019) 60:831–6. doi: 10.1093/jrr/rrz056 31423531 PMC6873619

[92] Sun J Zhang A Li W Wang Q Wang J Fan Y . CyberKnife stereotactic body radiation therapy as an effective treatment for hepatocellular carcinoma patients with decompensated cirrhosis. Front Oncol. (2020) 10:100. doi: 10.3389/fonc.2020.00100 32158688 PMC7052044

[93] Acker G Hashemi S-M Fuellhase J Kluge A Conti A Kufeld M . Efficacy and safety of CyberKnife radiosurgery in elderly patients with brain metastases: a retrospective clinical evaluation. Radiat Oncol. (2020) 15:1–10. doi: 10.1186/s13014-020-01655-8 32993672 PMC7523070

[94] Ryuno Y Abe T Iino M Saito S Aoshika T Oota T . High-dose stereotactic body radiotherapy using CyberKnife® for stage I peripheral lung cancer: a single-center retrospective study. Radiat Oncol. (2022) 17:128. doi: 10.1186/s13014-022-02094-3 35854333 PMC9297648

[95] Hayashi K Suzuki O Shiomi H Ono H Setoguchi A Nakai M . Stereotactic ablative body radiotherapy with a central high dose using CyberKnife for metastatic lung tumors. BMC Cancer. (2023) 23:215. doi: 10.1186/s12885-023-10635-6 36882702 PMC9990197

[96] Cheng X Li J Li Y Liu F Li T Meng Y . Efficacy of stereotactic body radiotherapy in hepatocellular carcinoma with adrenal metastases: a retrospective analysis. J Gastrointest Oncol. (2025) 16:1610–21. doi: 10.21037/jgo-2024-1011 40950326 PMC12432914

