# Supplementary material for: A glimpse into the CyberKnife robotic radiosurgery system: technical specifications and developments during thirty years
Source: Front Oncol. 2026 Jun 29;16:1871489. doi: 10.3389/fonc.2026.1871489 (PMC13357204; doi:10.3389/fonc.2026.1871489)
Supplement: Supplementary file 1 [file Table1.docx]

Supplementary Material

# Supplementary Table

**Supplementary Table 1**. A detailed description of changes and improvements applied to the CyberKnife system from 1987 to 2020.

|  | CyberKnife Neurotron 1000 in 1994 | CyberKnife System G3 in 2001 | CyberKnife System G4 in 2005 | CyberKnife VSI System in 2010 | CyberKnife System M6 in 2012 | CyberKnife S7 System in 2020 |
| --- | --- | --- | --- | --- | --- | --- |
| Target Tracking Method | 3D Skull skeletal tracking | 1. 6D Skull Tracking 2. Fiducial Tracking 3. Synchrony® Respiratory Tracking System | 1. 6D Skull Tracking 2. Fiducial Tracking 3. Synchrony® Respiratory Tracking System 4. Xsight™ Spine Tracking System 5. Xsight™ Lung Tracking System | 1. 6D Skull Tracking 2. Fiducial Tracking 3. Xsight® Spine Tracking 4. Xsight Lung Tracking 5. Synchrony® Respiratory Tracking System 6. InTempo™ Adaptive Imaging System 7. Supported Tracking Combinations | 1. 6D Skull Tracking 2. Fiducial Tracking 3. Xsight® Spine Tracking 4. Xsight Spine Prone Tracking (optional) 5. Xsight Lung Tracking (optional) 6. Lung optimized treatment: 1-view lung tracking and 0-view lung tracking (optional) 7. Synchrony® respiratory tracking system 8. InTempo™ Adaptive Imaging System (optional) | 1. 6D Skull Tracking 2. Fiducial Tracking 3. Xsight® Spine Tracking 4. Xsight Spine Prone Tracking (optional) 5. Xsight Lung Tracking (optional) 6. Lung optimized treatment: 1-view lung tracking and 0-view lung tracking (optional) 7. Synchrony® respiratory tracking system 8. InTempo™ Adaptive Imaging System (optional) |
| Roboter Manufactory | GMFanuc robotics corporation auburn hills MI | KUKA Systems GmbH, Augsburg, Germany | KUKA Systems GmbH, Augsburg, Germany (KR 240 KR C2) | KUKA Systems GmbH, Augsburg, Germany (KR 240-2) series 2000 | KUKA Systems GmbH, Augsburg, Germany KUKA QUANTEC KR300 R2500 Ultra robot | KUKA Systems GmbH, Augsburg, Germany KUKA QUANTEC KR300 R2500 Ultra robot |
| Robotic Arm | 1. 6-DOF robotic manipulator 2. Repeatability < ± 0. 5 mm | 1. 6-DOF robotic manipulator 2. Payload 250 kg 3. Repeatability < ± 0. 2 mm 4. Weight 1125 kg | 1. 6-DOF robotic manipulator 2. Payload 240 kg 3. Maximum Reach 2700 mm 4. Repeatability < ± 0. 12 mm 5. Weight 1267 kg | 1. 6-DOF robotic manipulator 2. Payload 240 kg 3. Maximum Reach 2700 mm 4. Repeatability < ± 0.12 mm 5. Weight 1267 kg | 1. 6-DOF robotic manipulator 2. Payload 300 kg 3. Maximum Reach 2500-3100 mm 4. Repeatability < ± 0.06 mm 5. Weight 1220 kg | 1. 6-DOF robotic manipulator 2. Payload 300 kg 3. Maximum Reach 2500-3100 mm 4. Repeatability < ± 0.06 mm 5. Weight 1220 kg |
| Static Targeting Accuracy | Mean: 1.6 mm  Range: 0.6 mm – 2.5 mm | Total clinical accuracy (0.95 mm) | 1. 6D skull Tracking = 0.44 ± 0.12 mm 2. Fiducial Tracking = 0.29 ± 0.10 mm 3. Xsight spine tracking = 0.53 ± 0.16 mm | The Maximum RMS total clinical accuracy less than 0.95 mm | The Maximum RMS total clinical accuracy less than 0.95 mm | The Maximum RMS total clinical accuracy less than 0.95 mm |
| Dynamic Targeting Accuracy | N/A | Synchrony accuracy specification = 1.5 mm | Synchrony accuracy specification = 1.5 mm | The Maximum RMS total clinical accuracy for Synchrony® respiratory tracking option less than 1.5 mm | The Maximum RMS total clinical accuracy for Synchrony® respiratory tracking option less than 1.5 mm | The Maximum RMS total clinical accuracy for Synchrony® respiratory tracking option less than 1.5 mm |
| Beam Collimation | Fixed circular collimators | Fixed circular collimators (5, 7.5, 10, 12.5, 15, 20, 25, 30, 35-, 40-, 50- and 60-mm diameter at 800 mm SAD) | 1. Fixed circular collimators (5, 7.5, 10, 12.5, 15, 20, 25, 30, 35-, 40-, 50- and 60-mm diameter at 800 mm SAD) 2. Iris Variable Aperture Collimator (5, 7.5, 10, 12.5, 15, 20, 25, 30, 35-, 40-, 50- and 60-mm diameter at 800 mm SAD) | 1. Fixed circular collimators (5, 7.5, 10, 12.5, 15, 20, 25, 30, 35-, 40-, 50- and 60-mm diameter at 800 mm SAD) 2. Iris Variable Aperture Collimator (5, 7.5, 10, 12.5, 15, 20, 25, 30, 35-, 40-, 50- and 60-mm diameter at 800 mm SAD) | 1. Fixed circular collimators (5, 7.5, 10, 12.5, 15, 20, 25, 30, 35-, 40-, 50- and 60-mm diameter at 800 mm SAD) 2. Iris Variable Aperture Collimator (5, 7.5, 10, 12.5, 15, 20, 25, 30, 35-, 40-, 50- and 60-mm diameter at 800 mm SAD) 3. InCise™ 2 Multileaf Collimator 4. CyberKnife M6 FI System (Fixed and Iris™ Variable Aperture Collimator) 5. CyberKnife M6 FM System (Fixed and InCise™ 2 Multileaf Collimator) 6. CyberKnife M6 FIM System (Fixed, Iris Variable Aperture and InCise™ 2 Multileaf Collimator) | 1. Fixed circular collimators (5, 7.5, 10, 12.5, 15, 20, 25, 30, 35-, 40-, 50- and 60-mm diameter at 800 mm SAD) 2. Iris Variable Aperture Collimator (5, 7.5, 10, 12.5, 15, 20, 25, 30, 35-, 40-, 50- and 60-mm diameter at 800 mm SAD) 3. InCise™ 2 Multileaf Collimator 4. CyberKnife S7 FI System (Fixed and Iris™ Variable Aperture Collimator) 5. CyberKnife S7 FM System (Fixed and InCise™ 2 Multileaf Collimator) 6. CyberKnife S7 FIM System (Fixed, Iris Variable Aperture and InCise™ 2 Multileaf Collimator) |
| Xchange® Robotic Collimator Changer | N/A | N/A | This system is included with the fixed and Iris™ Variable Aperture Collimator | This system is included with the fixed and second-generation Iris™ Collimator | This system is included with the fixed, second-generation Iris™ Variable Aperture and InCise™ 2 Multileaf Collimator | This system is included with the fixed, Iris™ Variable Aperture and InCise™ 2 Multileaf Collimator |
| Accelerator | 6 MV linear accelerator | 6 MV linear accelerator | 6MV X-band linear accelerator | 6 MV nominal photon energy | 6 MV nominal photon energy | 6 MV nominal photon energy |
| Dose-rate | 300 MU/min | 300-400 MU/min | 600-800 MU/ min | 800-1000 MU/min | 1000 MU/min | 1000 MU/min |
| Image detectors | Fluoroscopic screen/charge-coupled device camera (200*200) gadolinium oxysulfide fluoroscopes with pixel size 1.3 x 1.3 mm | Amorphous silicon x-ray screens (512*512) | High resolution flat-panel amorphous silicon with pixel size 0.4 * 0.4 mm (1024 × 1024) | High resolution flat-panel amorphous silicon with pixel size 0.4 * 0.4 mm (1024 × 1024) | High resolution flat-panel amorphous silicon with cesium iodide scintillator (1024 × 1024) | High resolution flat-panel amorphous silicon with cesium iodide scintillator (1024 × 1024) |
| Feature | 1. A compact X-band accelerator mounted on a robotic arm 2. A Frameless stereotaxic radiosurgery 3. An image-guided radiosurgery system | 1. A Frameless stereotaxic radiosurgery 2. An image-guided radiosurgery system 3. Patient-motion compensation during actual treatment 4. Supports multi-mode image fusion CT/MR/PET, DICOM RT/RTSS import/export supported 5. Multi treatment planning workstation option software designed as a workflow model 6. An automated beam correction | 1. Treat tumor anywhere in the body with sub-millimeter accuracy 2. Delivering non-invasive radiosurgery 3. Synchrony® Respiratory Tracking System 4. Continual Image Guidance throughout the Treatment 5. 4D Treatment Planning and Optimization System 6. Iris™ Variable Aperture Collimator 7. MultiPlan™ Treatment Planning System (CyRIS™) 8. Automated Non-Coplanar Treatment Delivery 9. Monte Carlo Dose Calculation 10. Contact Detection Sensor | 1. New Auto-Segmentation option for moving extracranial sites 2. Fast Treatment Planning 3. Iris™ Variable Aperture Collimator 4. Beam/time reduction techniques 5. 3D Advanced Visualization and rendering image 6. Support PET image data 7. New Tumor tracking methods 8. Ability to take medium resolution image 9. Support the Accuray Treatment Planning Services 10. Monte Carlo Dose Calculation | 1. Advanced system geometry 2. Enhanced design 3. Two Multiplan® Treatment Planning System 4. Sequential Optimization 5. 4D Treatment Optimization and Planning System 6. Monte Carlo Dose Calculation 7. Advances in Software optimization 8. Beam/time reduction techniques 9. Couch-based tracking method 10. High maneuverable robotic arm 11. InCise™ 2 Multileaf Collimator 12. Enhanced Image Review 13. Fast Treatment Planning | 1. Previous features of the M6 2. Fast installation, planning and delivery 3. Accurate, sub-millimeter, stereotactic treatments anywhere in the body 4. Delivery personalized treatments in-sync with target motion for every indication 5. Synchrony system and the VOLO™ Optimizer 6. Optimize treatment plans in as little as 60 seconds 7. Deliver motion-synchronized with sub-millimeter precision in as little as 15 minutes 8. New colored design (dark blue base, pink, blue and neutral gray) |
| Treatment Planning | Isocentric Planning | Isocentric Planning | 1. Non-coplanar and non-isocentric 2. Conformal Planning 3. Sequential Optimization | 1. Isocentric Planning 2. Conformal Planning | 1. Isocentric Planning 2. Conformal Planning 3. Sequential optimization Planning | 1. Isocentric Planning 2. Conformal Planning 3. Sequential optimization Planning 4. VOLO™ optimizer |
| Dose calculation algorithm(s) | Ray Tracing | Single, multi-center and conformal shape inverse-planning algorithms | 1. Monte Carlo Dose Calculation | 1. Ray Tracing Dose Calculation 2. Monte Carlo Dose Calculation | 1. Ray Tracing Dose Calculation 2. Monte Carlo Dose Calculation | 1. Ray Tracing Dose Calculation 2. Monte Carlo Dose Calculation |
| Workspace Robot | --- | --- | Workspace Robot: Right 64% and Left 36% | Workspace Robot: Right 64% and Left 36% | Workspace Robot: Right 50% and Left 50% | Workspace Robot: Right 50% and Left 50% |
| Treatment Couch | Adjusted manually | 5-DOF Standard treatment couch | 1. 5-DOF Standard treatment couch 2. 6-DOF RoboCouch Patient Positioning System | 1. 5-DOF Standard treatment couch 2. 6-DOF RoboCouch Patient Positioning System | 1. 5-DOF Standard treatment couch 2. 6-DOF RoboCouch Patient Positioning System | 1. 5-DOF Standard treatment couch 2. 6-DOF RoboCouch Patient Positioning System |
| Treatment Table Top | N/A | N/A | 1. Flat tabletop (standard) 2. Seated Load table top (optional) | 1. Flat tabletop (standard) 2. Flat with RoboCouch® System 3. Seated Load table top (optional) | 1. Flat tabletop (standard) 2. Flat with RoboCouch® System 3. Seated Load table top (optional) | 1. Flat tabletop (standard) 2. Flat with RoboCouch® System 3. Seated Load table top (optional) |
| Clinical applications | Intracranial and upper spine radiosurgery | Intracranial, head, neck, lung and liver | Intracranial, spine, lung, liver, prostate and pancreas | Intracranial, head, neck, spine, paraspinal, lung, prostate, liver and pancreas | Intracranial, head, neck, spine, lung, prostate, liver and pancreas | Anywhere in the body including the prostate, lung, brain, neck, spine, liver, pancreas and kidney |
| Treatment time for SRS and SBRT treatment | --- | 45–60 minutes | 45–60 minutes | 25–30 minutes | 20–30 minutes | Less than 20 minutes |
| MU/min: Monitor Units per minute, N/A: not applicable, 6D: Six-degree, 6-DOF: Six-Degrees of Freedom, RMS: root mean square, SAD: source to axis distance, SRS: stereotactic radiosurgery, SBRT: stereotactic body radiation therapy | | | | | | |

.
